# Identification and Expression Analysis of Snf2 Family Proteins in Tomato (*Solanum lycopersicum*)

**DOI:** 10.1155/2019/5080935

**Published:** 2019-03-28

**Authors:** Dongdong Zhang, Sujuan Gao, Ping Yang, Jie Yang, Songguang Yang, Keqiang Wu

**Affiliations:** ^1^Key Laboratory of South China Agricultural Plant Molecular Analysis and Genetic Improvement, Guangdong Provincial Key Laboratory of Applied Botany, South China Botanical Garden, Chinese Academy of Sciences, Guangzhou 510650, China; ^2^University of Chinese Academy of Sciences, Chinese Academy of Sciences, Beijing 100049, China; ^3^College of Light Industry and Food Science, Zhongkai University of Agriculture and Engineering, Guangzhou 510225, China; ^4^Institute of Plant Biology, National Taiwan University, Taipei 106, Taiwan

## Abstract

As part of chromatin-remodeling complexes (CRCs), sucrose nonfermenting 2 (Snf2) family proteins alter chromatin structure and nucleosome position by utilizing the energy of ATP, which allows other regulatory proteins to access DNA. Plant genomes encode a large number of Snf2 proteins, and some of them have been shown to be the key regulators at different developmental stages in *Arabidopsis*. Yet, little is known about the functions of Snf2 proteins in tomato (*Solanum lycopersicum*). In this study, 45 Snf2s were identified by the homologous search using representative sequences from yeast (*S. cerevisiae*), fruit fly (*D. melanogaster*), and *Arabidopsis* (*A. thaliana*) against the tomato genome annotation dataset. Tomato Snf2 proteins (also named SlCHRs) could be clustered into 6 groups and distributed on 11 chromosomes. All SlCHRs contained a helicase-C domain with about 80 amino acid residues and a SNF2-N domain with more variable amino acid residues. In addition, other conserved motifs were also identified in SlCHRs by using the MEME program. Expression profile analysis indicated that tomato Snf2 family genes displayed a wide range of expressions in different tissues and some of them were regulated by the environmental stimuli such as salicylic acid, abscisic acid, salt, and cold. Taken together, these results provide insights into the functions of SlCHRs in tomato.

## 1. Introduction

In eukaryotes, about 147 bp of DNA wrapping around a histone octamer forms nucleosome, the fundamental unit of chromatin. The reversible changes in chromatin structure alter the stability of the nucleosome, thereby facilitating regulatory factors access, such as transcription factor [1, 2]. Thus, the precise chromatin structure is essential for the correct spatial and temporal gene expression in the eukaryotes [3, 4]. The changes in chromatin involve histone modifications, DNA methylation, histone variants, and chromatin remodeling. Many proteins have been identified to mediate these processes, among which the Snf2 family proteins can affect gene expression by using the energy of ATP hydrolysis to alter the interactions between histones and DNA [5]. Indeed, most Snf2 proteins associated with other chromatin remodelers form large multisubunit complexes called chromatin-remodeling complexes, which most likely alter the activity of the core ATPase *in vivo*. The accessory subunits commonly contain additional domains that may affect the enzymatic activity of the complex, facilitate its binding to other proteins, and target the complex to DNA and/or modified histones [6]. The chromatin-remodeling complexes are conserved throughout eukaryotes with essential roles in many aspects of chromatin biology.

Based on the different protein compositions and functions, the chromatin-remodeling complexes can be divided into SWI/SNF, ISWI (imitation switch), INO80 (inositol requiring 80), and CHD (chromodomain, helicase, and DNA binding) groups [7]. The SWI/SNF complexes alter the position of the nucleosome at promoters, which can regulate transcription either positively or negatively [8]. The ISWI group complexes were essential for chromatin assembly and the formation of nucleosome arrays with well-ordered spacing, which might help to promote repression [9]. In yeast, the *ino80* mutants are defects in homologous recombination during DNA repair, indicating that the INO80 complexes are involved in DNA repair [10]. Indeed, the INO80 complexes could be recruited to double-stranded breaks (DSBs) via direct binding of the complex subunits to phosphorylated H2AX or *γ-*H2AX [11, 12], which facilitates nucleosome eviction at DSBs, allowing the recruitment of repair factors. In comparison, the CHD complexes have diverse functions. For instance, CHD1 is targeted to sites of active transcription through PHD-mediated recognition of H3K4me3 [13, 14] and associates with other preinitiation factors to facilitate transcriptional elongation and splicing [15]. In addition, CHD3 and CHD4 are incorporated into a large protein complex with histone deacetylases to repress transcription by binding to methylated DNA in an MBD2/3-dependent manner, remodeling the surrounding chromatin, and removing active histone marks [16, 17].

SWI2/SNF2, the first Snf2 protein, was identified from *Saccharomyces cerevisiae* by the examination of mating type switching (SWI) and sucrose nonfermenting (SNF) mutants [18]. Further data indicated that the *SWI2*/*SNF2* gene is homologous to a number of other ATP-binding helicases of the DEAD/H family [19]. The sequence similarity includes the catalytic ATPase domain and seven characteristic protein motifs [20]. Moreover, the conserved domain analysis indicated that the helicase-like region can be further divided into two domains: the SNF2-N and helicase-C domains [21, 22]. Based on the helicase-like region, the Snf2 family proteins are grouped into six clades, including the Snf2-like, SWI/SNF-related protein-like (Swr1-like), Rad54-like, Rad5/16-like, SSO1653-like, and Distant family [21].

*Arabidopsis* contains 41 Snf2 family proteins that fall into 18 subfamilies [22]. The function of *Arabidopsis* BRAHMA (BRM) and SPLAYED (SYD), the closest homologs of yeast and animal SWI2/SNF2 ATPase subunits (Snf2 subfamily), has been investigated. Mutations of *SYD* cause defects of the shoot apical meristem (SAM). Furthermore, SYD physically interacts with the promoter of *WUSCHEL* (*WUS*), a central regulator in SAM [23]. The expression profile showed that *BRM* was mainly expressed in the active cell division tissues, such as meristems and organ primordia [24]. The BRM mutants displayed multiple developmental defects, such as reduced plant size and root length [24, 25], downward curling leaves [25], more sensitivity to abscisic acid (ABA) [26], and early flowering [27]. SYD and BRM were shown to interact with LEAFY and SEPALLATA3 proteins, which are essential for floral organ identity [28]. Indeed, the functions of BRM to modulate gene transcription are always through association with other nuclear proteins. For example, the plant-unique H3K27 demethylase, RELATIVE OF EARLY FLOWERING 6 (REF6), recruits BRM to its target genomic loci containing a CTCTGYTY motif [29]. Moreover, FORGETTER1 (FGT1), which is specifically required for the heat stress memory coactivator, maintains its target loci in an open and transcription-competent state by interacting with BRM near the transcriptional start site [30]. BRM also interacts with other transcription factors such as TEOSINTE BRANCHED1 CYCLOIDEA AND PCF-CODING GENE (TCP4), ANGUSTIFOLIA3 (AN3), and BREVIPEDICELLUS (BP) to regulate gene expression involved in leaf development and inflorescence architecture [31, 32]. A recent report showed that BRM also interacts with PHY-INTERACTING FACTOR 1 (PIF1) to modulate *PROTOCHLOROPHYLLIDE OXIDOREDUCTASE C* (*PORC*) expression, which is essential for chlorophyll biosynthesis during the transition from heterotrophic to autotrophic growth [33]. Meanwhile, SUMOylation of BRM caused by METHYL METHANE SULFONATE SENSITIVITY 21 (MMS21) increases the BRM stability in root development [34]. Interestingly, more recent data demonstrated that microRNA precursors (pri-miRNAs) are the substrates of BRM. As a partner of the microprocessor component SERRATE (SE), BRM accesses pri-miRNAs through SE and remodels their secondary structures, which prevents further downstream processing mediated by DCL1 and HYL1 [35].

Transgenic *Arabidopsis* plants overexpressing *AtCHR12*, a member of the *Snf2* subfamily, exhibit growth arrest of primary buds and growth reduction of the primary stem under drought and heat stress [36]. Moreover, a Rad54-like family member, DEFECTIVE IN RNA-DIRECTED DNA METHYLATION1 (DRD1), and a member of Snf2-like protein, DECREASED DNA METHYLATION 1 (DDM1), are involved in DNA methylation [37, 38]. In addition, DRD1 and DDM1 are also involved in leaf senescence, since *drd1* and *ddm1* mutants exhibit a delayed leaf senescence phenotype [39]. Furthermore, PHOTOPERIOD-INDEPENDENT EARLY FLOWERING 1 (PIE1), a Swr1 subfamily member, known to deposit histone H2A.Z, is also important for flowering and plant development [40, 41], while the Mi-2 subfamily member PICKLE is a key regulator in brassinosteroid (BR), gibberellin (GA), and cytokinin (CK) signaling [42, 43].

Compared with *Arabidopsis*, little is known about Snf2 proteins in other plant species. In rice, *OsDDM1a* and *OsDDM1b*, two genes homologous to *Arabidopsis DDM1*, are involved in DNA methylation [44], while rice CHR729, a member of the CHD3 family, plays an important role in seedling development via the GA signaling pathway [45]. A previous study has also analyzed the DRD1 and Snf2 subfamilies in tomato, which were reported to be involved in stress responses [46]. In addition, constitutively overexpressing a *Snf2* gene (termed as *SlCHR1*, *Solyc01g079690*) caused reducing growth of transgenic tomato plants (cv. Micro-Tom) [47]. However, the largest and most diverse gene family, the *Snf2* gene family, has not been systematically analyzed in the tomato genome. In this study, we identified and characterized 45 Snf2 family proteins from tomato. The expression profiles of the tomato *Snf2* genes were also analyzed. The results provide a wealth of information for further exploring the developmental function of Snf2 family proteins in tomato, especially during fruit development.

## 2. Materials and Methods

### 2.1. Plant Materials and Growth Conditions

In this study, the *Solanum lycopersicum* cultivar “Heinz 1706” was used as an experimental material. Surface-sterilized tomato seeds were grown in the Murashige and Skoog (MS) medium with 1.5% sucrose and 0.8% agar for 14 days in a controlled environment greenhouse with a long photoperiod (16 h light/8 h dark) at 23 ± 1°C.

### 2.2. Identification of Tomato SlCHR Genes

The protein sequences of AtCHRs from *Arabidopsis thaliana*, *S. cerevisiae*, and *D. melanogaster* were retrieved from ChromDB (http://www.chromdb.org). The deduced sequences of SlCHR proteins in tomato were obtained as described elsewhere using the BLASTP program (https://solgenomics.net/tools/blast/, ITAG3.20). Then, the candidates of SlCHR proteins were confirmed using Pfam (http://pfam.xfam.org/) and SMART (http://smart.embl-heidelberg.de/) programs. The domain architecture was drawn using the DOG2.0 software [48].

### 2.3. Chromosome Location and Sequence Feature Analyses

Chromosome location of SlCHR genes was determined by BLAST analysis of SlCHRs against SGN (http://solgenomics.net/organism/Solanum_lycopersicum/genome). The program DnaSP was used to carry out synonymous substitution (Ks) values of paralogous gene pairs [49]. The Compute pI/Mw tool on the ExPASy server (http://web.expasy.org/compute_pi/) was used to predicted molecular weight (Mw) and theoretical isoelectric point (pI) of SlCHRs. The structures of SlCHR genes were predicted using the Gene Structure Display Server (http://gsds.cbi.pku.edu.cn/) [50].

### 2.4. Phylogenetic Construction and Motif Analysis

The phylogenetic trees were generated as described elsewhere using MEGA5.2 program [51]. The Pfam program (http://pfam.xfam.org/) and Conserved Domain Database (CDD, http://www.ncbi.nlm.nih.gov/Structure/cdd/wrpsb.cgi) were used to predict the conserved domains of SlCHRs. The 80 amino acids of the helicase-C domain were aligned with ClustalW. Sequence logos were generated using the WebLogo platform (http://weblogo.berkeley.edu/). Potential protein motifs were predicted using the MEME package (http://meme-suite.org/tools/meme).

### 2.5. Expression Data Visualization

The expression data of tomato SlCHRs were extracted from publicly available RNA-seq datasets from the Tomato Genome Consortium [52] and visualized with Matrix2PNG (http://www.chibi.ubc.ca/matrix2png/bin/matrix2png.cgi) [53]. The RNA-seq data were obtained from transcriptome sequencing using three-week-old sand-grown seedlings, roots, leaves, buds (unopened flower buds), and flowers (fully open flowers) as well as fruits (at 1 cM, 2 cM, and 3 cM), MG (mature green), breaker (B, early ripening), and 10-day post-B (B10, red ripe) stages of tomato “Heinz 1706.”

### 2.6. Gene Expression Analyses

For hormone and salt stress response test, 2-week-old tomato “Heinz 1706” seedlings grown in the MS medium were transferred to the liquid MS medium containing SA (1 mM), ABA (50 *μ*M), and NaCl (200 mM) for 4 h, respectively. For cold stress test, the planes were transferred to a 4°C growth cabinet for 4 h. Total RNA from treated seedlings was extracted with TRIzol reagent (Invitrogen) according to the manufacturer's protocol and used to synthesize cDNA. Real-time PCR was performed with iTaq™ Universal SYBR® Green Supermix (Bio-Rad) using ABI 7500 Fast Real-Time PCR System. The gene-specific primers for real-time PCR were designed by Primer 3.0 [36] and listed in Supplemental Table 2. Tomato Actin (Solyc03g078400) was served as an internal control.

## 3. Results

### 3.1. Identification of Snf2 Family Proteins in Tomato

To uncover the complete family of genes for encoding Snf2 proteins in the tomato genome, iterative BLASTP researches using representative sequences from yeast (*S. cerevisiae*), fruit fly (*D. melanogaster*), and *Arabidopsis* (*A. thaliana*) were conducted against SGN (http://solgenomics.net/organism/Solanum_lycopersicum/genome, ITAG3.20) genome annotation database. In total, 45 nonredundant putative *Snf2* genes were identified in the tomato genome (Table 1).

**Figure 1:**
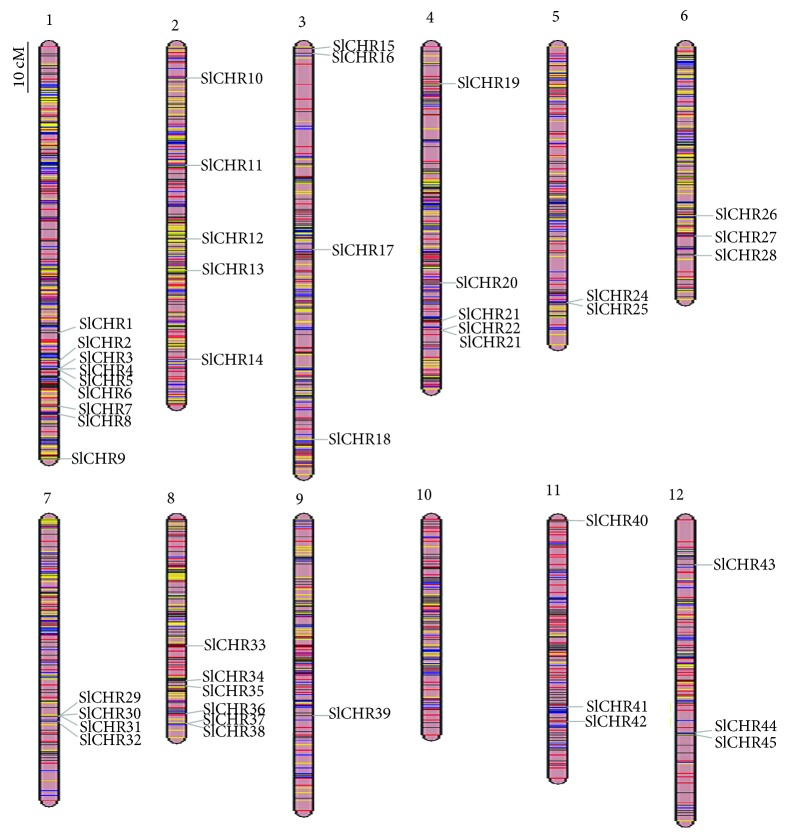
Chromosomal location of *SlCHR* genes. The scale represents 10 centimorgans.

According to the current used nomenclature in *Arabidopsis* and rice, we designated Snf2 proteins of tomato (*Solanum lycopersicum*) as SlCHRs. All of the deduced SlCHR proteins contained the conserved SNF2-N domain and helicase-C domain. The theoretical isoelectric point (pI) of SlCHR candidates ranged from 5.13 to 9.42, and the length of SlCHRs varied from 391 to 2500 amino acids. The molecular weight (Mw) and the number of introns varied from 44.3 to 274.2 kDa and 1 to 37, respectively (Supplemental Table 1). Mapping *SlCHRs* to the tomato genome showed that 45 *SlCHRs* were unevenly distributed on 11 chromosomes (except for chromosome 10). Among them, there were 9 SlCHRs on Chr1; 5 on each of Chr2 and Chr4; 3 on each of Chr6, Chr11, and Chr12; 4 on each of Chr3 and Chr7; 2 on Chr5; 6 on Chr8; and one on Chr9, respectively (Figure 1). Most *SlCHRs* were located in the bottom regions of tomato chromosomes, and few were in the central regions of chromosomes (Figure 1).

Moreover, 8 pairs of *SlCHRs* (Ks < 1.0) were evolved from intrachromosomal duplication (Supplemental Figure 1 and Table 2), indicating the importance of gene duplication for *SlCHR* gene expansion.

### 3.2. Phylogenetic Analysis of Snf2 Proteins in Tomato, Yeast, Fruit Fly, and *Arabidopsis*

**Figure 2:**
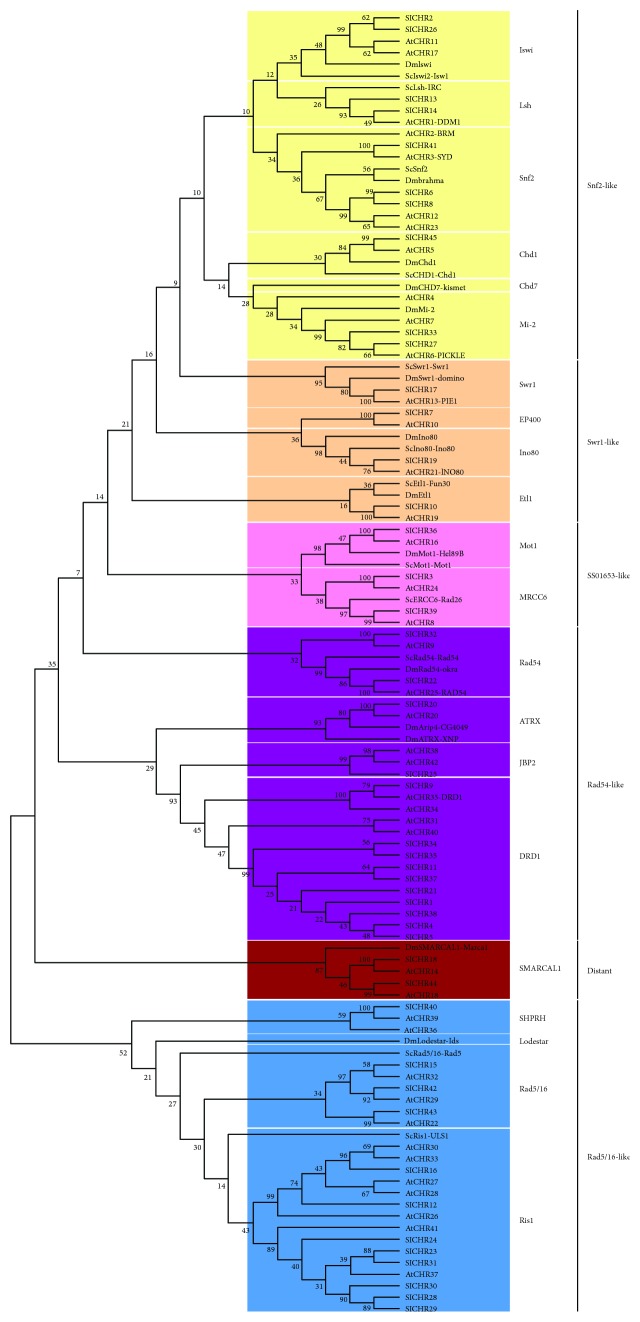
Neighbor-joining (NJ) phylogenetic tree for Snf2s in *S. cerevisiae* (Sc), *D. melanogaster* (Dm), *A. thaliana* (At), and *Solamum lycopersicum* (Sl). The groups of homologous genes identified and bootstrap values are shown. The reliability of branching was assessed by the bootstrap resampling method using 1,000 bootstrap replicates.

In order to investigate the evolutionary relationship of Snf2 proteins in tomato, *Arabidopsis* (*A. thaliana*), yeast (*S. cerevisiae*), and fruit fly (*D. melanogaster*), a neighbor-joining (NJ) phylogenetic tree was constructed with 45 SlCHRs, 30 AtCHRs, 13 ScSnf2s, and 14 DmSnf2s using MEGA5.2. The results showed that the 45 SlCHR proteins were grouped into 6 clusters, namely, the Snf2-like (10 members), Swr1-like (4 members), SSO1653-like (3 members), Rad54-like (14 members), Distant family (2 members), and Rad5/16-like (12 members). Additionally, each subfamily could be further divided into subgroups. For example, Rad54-like subfamily was further classified into four subgroups, namely, Rad54, J-binding protein 2 (JBP2), alpha thalassemia/mental retardation syndrome X-linked (ATRX), and DRD1, containing 2 (SlCHR22 and SlCHR32), 1 (SlCHR25), 1 (SlCHR20), and 10 SlCHRs, respectively (Figure 2 and Table 1). Tomato possessed 10 proteins belonging to the Snf2-like subfamily, which fell into the chromodomain, helicase, and DNA binding (Chd1) (1 member); Mi-2 (2 members); Imitation SWI2 (Iswi) (2 members); lymphoid-specific helicase (Lsh) (2 members); and Snf2 (3 members) subgroup, respectively (Figure 2 and Table 1).

Phylogenetic analysis showed that SlCHR6, SlCHR41, and SlCHR8 (also named as SlCHR1 in a recent report) displayed high sequence homology with Scsnf2 and DmBrahma, the ATPases of SWI/SNF-type chromatin-remodelingcomplex in yeast and fruit fly (Figure 2). In addition, 7 sister pairs (SlCHR2-SlCHR26, SlCHR6-SlCHR8, SlCHR34-SlCHR35, SlCHR11-SlCHR37, SlCHR4-SlCHR5, SlCHR23-SlCHR31, and SlCHR28-SlCHR29) were very likely to be paralogous proteins (Figure 2), while 20 pairs of SlCHRs seemed to be orthologous proteins (Figure 2). Among these paralogous proteins, SlCHR2/26 and SlCHR6/8 belonged to the Snf2-like subfamily and SlCHR34/35, SlCHR11/37, and SlCHR4/5 were from the Rad54-like subfamily, whereas SlCHR23/31 and SlCHR28/29 were in the Rad5/16-like subfamily (Figure 2). The wider paralogous pairs existed in SlCHR proteins, indicating that the expansion of *SlCHR* genes occurred after separation of paralogous genes. Interestingly, in the unrooted phylogenetic tree based on the data from *Arabidopsis*, rice, and tomato (Supplemental Figure 2), two distinct branches in the DRD1 subfamily and Ris1 subfamily were consisted of only SlCHRs. These data indicated that expansion of DRD1 and Ris1 members in tomato was most like due to gene duplication.

### 3.3. Comparative Analysis Gene Structures of *SlCHR* and *AtCHR*

**Figure 3:**
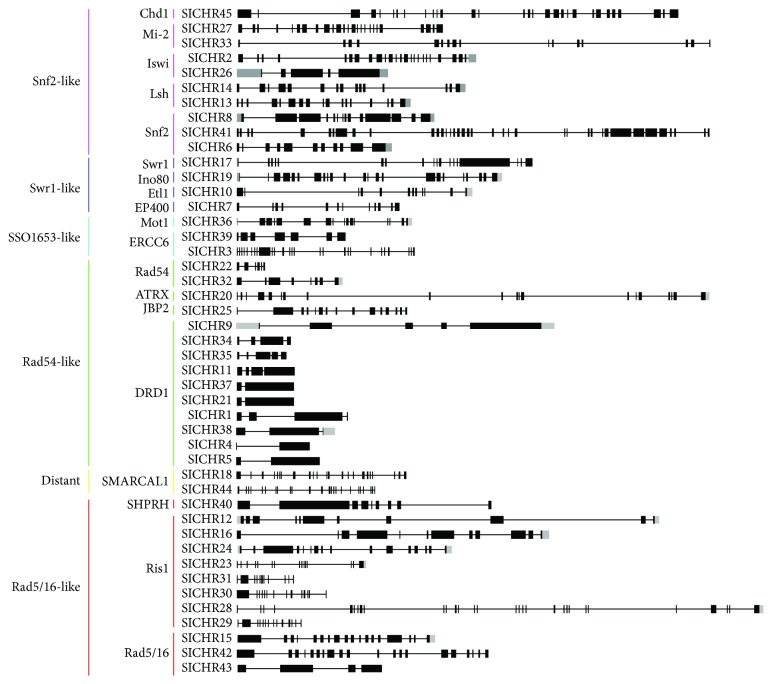
Exon-intron structures of *SlCHR* genes. Introns are represented by lines. Exons are indicated by green boxes, while UTR is indicated by gray boxes.

Gene structure analysis of 45 *SlCHR* genes displayed that the number of introns varied from 1 (*SlCHR21*, *SlCHR4*, *SlCHR5*, and *SlCHR21*) to 37 (*SlCHR41*) (Figure 3 and Supplemental Table 1). By contrast, the intron number of 41 *AtCHRs* varied between 2 and 33 (Supplemental Table 1 and Supplemental Figure 3). The length of introns also varied significantly among the *SlCHR* subfamily including Snf2-like, Swr1-like, and Rad5/16-like genes (Figure 3). Interestingly, the distribution of intron phases in *SlCHRs* was very similar to *AtCHRs* (Supplemental Figure 4).

**Figure 4:**
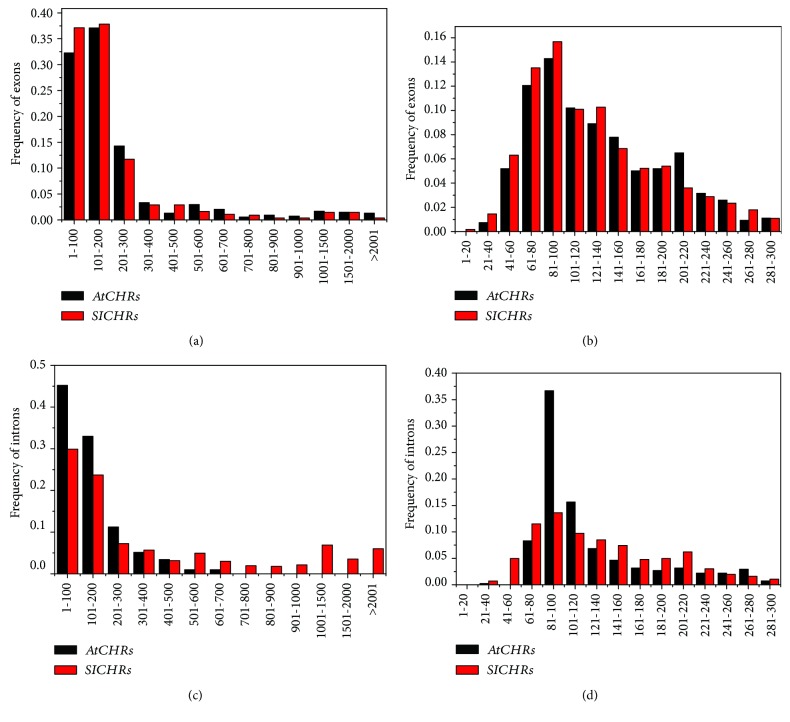
Size distribution of exons and introns in *AtCHRs* and *SlCHRs*. (a) Size distribution of exons in *AtCHRs* and *SlCHRs*, (b) detailed size distribution of small exons in *AtCHRs* and *SlCHRs*, (c) size distribution of introns in *AtCHRs* and *SlCHRs*, and (d) detailed size distribution of small introns in *AtCHRs* and *SlCHRs*.

Next, we compared the internal exons and introns of *SlCHRs* with those of *AtCHRs*. The results showed that the exons of *SlCHRs* varied 18 to 3067 bp with the average of 215 bp, which was smaller than the average length of *AtCHR* exons (263 bp). Interestingly, most CHRs (about 86% of *SlCHR* and 83% of *AtCHR*) had an exon with a size below 300 bp (Figure 4(a)), while 56% of *SlCHR* exons and 53% of *AtCHR* exons were between 60 and 160 bp (Figure 4(b)).

Although the size distribution of *SlCHR* exons was similar to *AtCHR* exons, the size distribution of intron was more variable, ranging from 34 bp to 9.0 kb. There were 54 *SlCHR* introns (9.5%) with sizes > 1.5 kb; however, no such introns existed in *AtCHRs* (Figure 4(c)). About 61% of *SlCHR* and 89% of *AtCHR* introns had sizes below 300 bp, while 56% of *SlCHR* introns were between 60 and 160 bp and 53% of *AtCHR* introns were between 80 and 120 bp, respectively (Figure 4(c)). Meanwhile, the average sizes of *SlCHR* introns and *AtCHRs* were 595 bp and 153 bp, respectively. These results indicated that the exon and intron size distribution was different between *SlCHRs* and *AtCHRs*.

### 3.4. The Conserved Motifs in SlCHRs

**Figure 5:**
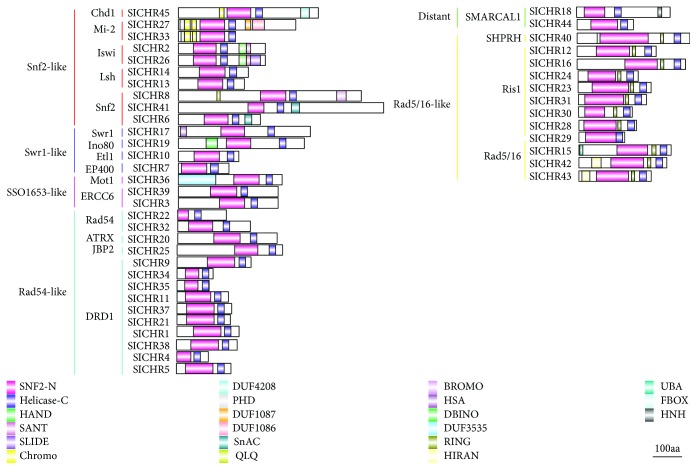
Domain architectures of tomato Snf2 family proteins. Different domains are showed by a rectangle with different colors and numbers. The scale represents the length of the protein and all proteins are displayed in proportion.

To investigate the conserved domains of SlCHRs, Pfam (http://pfam.xfam.org/) and Conserved Domain Database (CDD, http://www.ncbi.nlm.nih.gov/Structure/cdd/wrpsb.cgi) programs were used. The results showed that all the 45 SlCHRs contained a helicase-C domain with about 80 amino acid residues and a SNF2-N domain with more variable amino acid residues (Figure 5).

Unlike the human Snf2 subfamily proteins hBRG1and hBRM, the conserved HSA (helicase-SANT-associated) domain was not found in all three Snf2 subfamily proteins (SlCHR8, SlCHR41, and SlCHR6) and only SlCHR8 contained bromodomain, an acetyl-lysine binding domain (Figure 5). However, an alignment profile using the HSA domain of humans and the N-termini of SlCHR8, SlCHR41, and SlCHR6 showed that the conserved amino acid residues including E, H, and L were found in tomato Snf2 subfamily proteins (Supplemental Figure 5). Interestingly, the Swr1 subfamily SlCHR17 was highly homologous to *Arabidopsis* PIE1, containing the HSA domain at the N-terminus (Figure 5). Furthermore, two members of the Iswi subfamily, SlCHR2 and SlCHR26, had the conserved domains HAND, SANT, and SLIDE located on the C-terminus (Figure 5). The Mi-2 subfamily proteins, SlCHR27 and SlCHR33, contained two double chromodomains and an additional PHD domain at the N-terminal part of the proteins. All members of the Rad5/16-like family group except SlCHR29 had a RING-finger E3 ubiquitin ligase domain embedded between the SNF2-N and helicase-C domain in the C-terminal regions (Figure 5). Furthermore, an additional HIRAN domain was found in the N-terminal region of SlCHR42 and SlCHR43 in this group. In general, the HIRAN domain was predicted to recognize features associated with damaged DNA or stalled replication forks, such as ssDNA stretches or DNA lesions [54].

In addition to these conserved domains, other conserved motifs were searched using the MEME program. 20 motifs for 45 SlCHRs were identified (Table 3). The number of motifs in each SlCHR varied from 5 to 16 (Table 3). Motifs 10, 4, and 1 were actually the helicase-C domain (Supplemental Figure 6) that was found in most of the SlCHRs. In addition to the conserved motifs, several other motifs were also identified in SlCHR proteins, such as motifs 13, 16, 20, 7, 8, and 18 in the DRD1 subfamily as well as motifs 17, 14, 15, and 19 in the Rad5/16-like group (Table 3). Sequence analysis of helicase-C domains identified the conserved acid residues such as Asp, Gly, Arg, Gln, and Lue in the motifs 10, 4, and 1 (Supplemental Figure 6 and Supplemental Figure 7).

### 3.5. Expression Patterns of Tomato *Snf2* Family Genes

**Figure 6:**
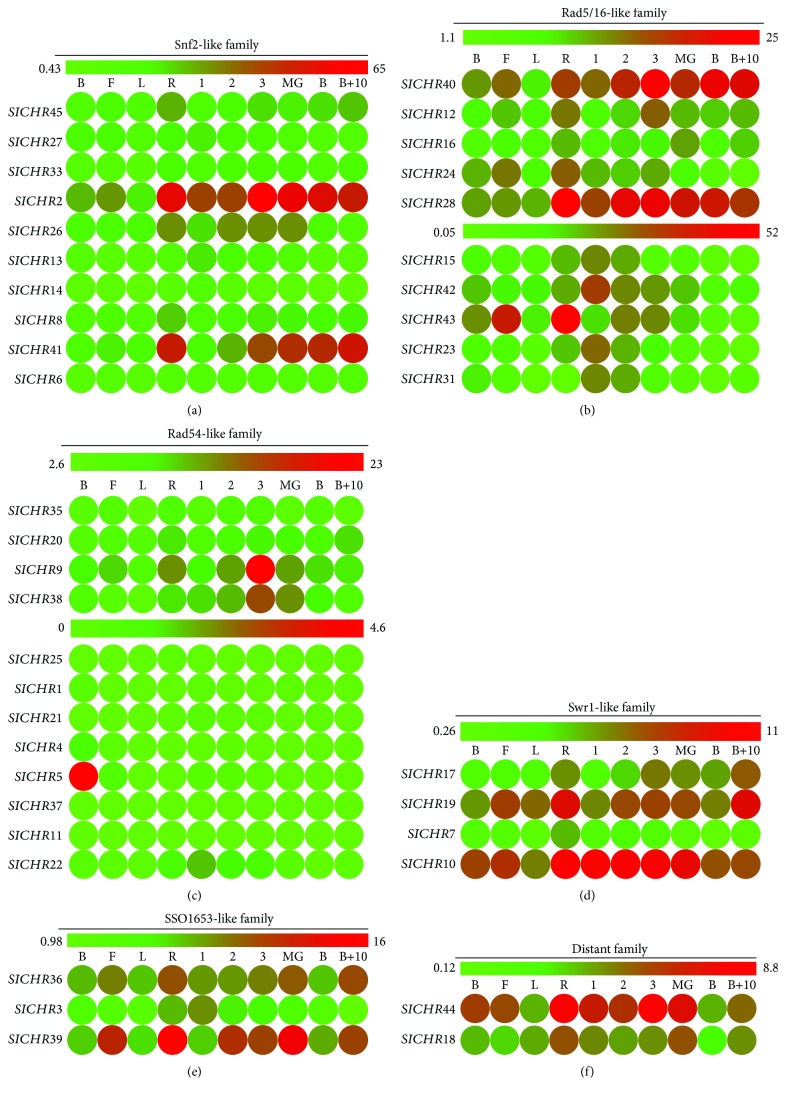
Expression profiles of tomato Snf2s. Heat map of RNA-seq expression data from bud (B), flower (F), leaf (L), root (R), 1cM_fruit (1), 2cM_fruit (2), 3cM_fruit (3), mature green fruit (MG), berry at breaker stage (B), and berry ten days after breaking (B+10). The expression values are measured as reads per kilobase of the exon model per million mapped reads (RPKM).

In order to explore the possible role of tomato *snf2s*, we analyzed their expression profiles (Figure 6). *SlCHR2*, *SlCHR26*, *SlCHR8*, and *SlCHR41* (Snf2-like family) had similar expression profiles and were expressed mainly in roots and fruits from 1 cM to B stages (Figure 6(a)), suggesting that these genes may play redundant roles in root and fruit development. *Swr1*-like *SlCHRs*, *SlCHR17*, and *SlCHR19* were strongly expressed in roots and B+10 stage fruits, while *SlCHR7* was mostly expressed in roots (Figure 6(b)). Interestingly, *SlCHR10* was expressed in roots and in the early stages of fruit development (Figure 6(b)). Most of the *SSO1653-like* and *Distant SlCHR* genes accumulated in the early stages of fruit development and roots (Figures 6(d) and 6(f)). According to the expression profile of *Rad54-like* and *Rad5/16-like SlCHRs*, these *SlCHRs* could be categorized into two groups: high expression and low expression (Figures 6(c) and 6(e)). However, some *SlCHR* genes showed specific expression peaks. For example, *SlCHR9*, *SlCHR38*, and *SlCHR40* were strongly expressed in fruits at the 3 cM stage, *SlCHR4* and *SlCHR5* in buds, while *SlCHR28* and *SlCHR43* in roots (Figures 6(c) and 6(e)). In contrast, *SlCHR30*, *SlCHR34*, and *SlCHR35* were not detected in all tissues analyzed (Figure 6).

**Figure 7:**
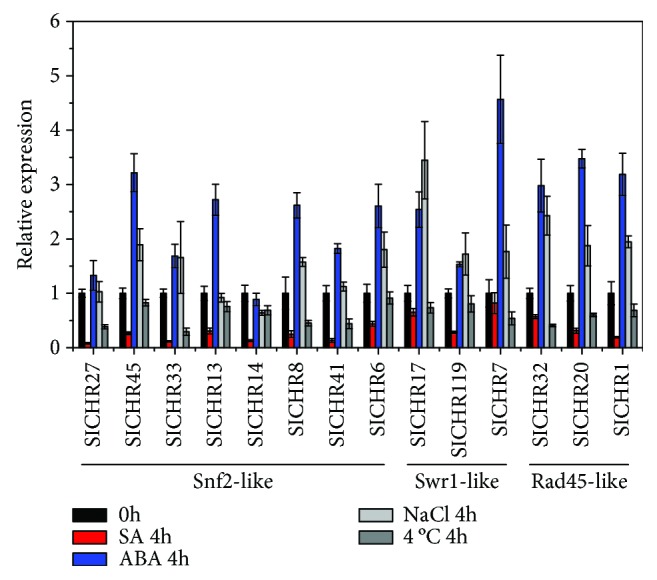
Expression profile of *SlCHRs* responding to hormones, salt, and cold tested by RT-PCR. Seedlings of two-week-old plate-cultured plants were treated with SA (1 mM), ABA (50 *μ*M), NaCl (200 mM), and cold (4°C) for 4 h and collected for total RNA isolation. RT-PCR was amplified using gene-specific primers. The tomato *Actin* (*Solyc03g078400*) was used as an internal control. Error bars indicate the SE. The same results were obtained in two independent experiments.

We further investigated the expression pattern of *SlCHR* genes responding to environmental stimuli including hormones, salt, and cold by qRT-PCR. All of the genes analyzed were clearly repressed by SA and cold treatment, especially *SlCHR27* (Figure 7). Most of the genes analyzed except *SlCHR14* were induced by ABA and salt treatments. In particular, *SlCHR7* and *SlCHR17* were strongly induced by ABA and salt treatment, respectively (Figure 7). These results revealed that these *SlCHR* genes may be involved in response to different environmental stimuli in tomato.

## 4. Discussion

Snf2 family proteins are the catalytic subunit of the ATPase chromatin-remodeling complexes and contain highly conserved SNF2-N (DEAD) and helicase-C (HELICs) domains involved in many aspects of DNA events such as transcription, replication, homologous recombination, and DNA repair [6, 7]. In this study, we systematically identified 45 genes encoding Snf2 proteins (SlCHRs) in tomato (*Solanum lycopersicum*), which are distributed on 11 chromosomes (Table 1 and Figure 1). Eight pairs of *SlCHR* intrachromosomal duplication were identified, indicating that gene duplication may play an important role in *SlCHR* gene expansion in tomato (Table 2 and Supplemental Figure 1). Similar results were also reported in other organisms such as human and *Arabidopsis* [55, 56]. The intron phases were similar in *SlCHRs* and *AtCHRs* (Supplemental Figure 3), indicating that plant *Snf2* genes originate from a common ancestor. Previously, a number of genes encoding Snf2 proteins have been identified in *Arabidopsis* [22], rice [57], and tomato [46]. Nevertheless, only the members of DRD1 and Snf2 subfamilies were identified in tomato previously [46]. Consistent with the previous report, 3 members of Snf2, SlCHR8 (Solyc01g079690), SlCHR41 (Solyc11g062010), and SlCHR6 (Solyc01g094800), were identified. In addition, other three members, SlCHR34, SlCHR35, and SlCHR40, belonging to the DRD1 subfamily, were also found (Table 1).

Sequence comparative analysis of tomato *SlCHRs* and *Arabidopsis AtCHRs* revealed some conserved features. For example, all deduced CHRs contained the highly conserved helicase-C domain with about 80 amino acid residues (Figure 5, Supplemental Figure 6 and Supplemental Figure 7). Unlike the members of the human Snf2 subfamily, the three Snf2 subfamily proteins (SlCHR8, SlCHR41, and SlCHR6) in tomato lack the conserved HSA domain (Figure 5). Nevertheless, like the HSA domain of yeast and human Snf2 proteins, the N-terminal of tomato Snf2 CHRs also has the conserved amino acid residues E, H, and L (Supplemental Figure 5). As the primary binding platform for nuclear actin-related proteins (ARPs) and actin, the HSA domain is important for the activity of chromatin-remodeling ATPases in yeast and animals [58]. Indeed, the ARPs are conserved subunits of the SWI/SNF and INO80 chromatin-remodeling complexes that associate directly with the ATPase via the HSA domain [58]. The bromodomain was first identified in BRM, the *Drosophila* homolog of SWI2/SNF2, binding acetylated residues on histone tails [59]. Therefore, SlCHR8 may be the ATPase of at least one of the putative SWI/SNF complexes in tomato. Additional domains such as HAS and SANT that facilitate interaction with the other proteins, as well as bromodomain, chromodomain, and PHD domains that modified histones, were also found in SlCHRs (Figure 5).

Previous reports showed that *AtCHRs* played key roles in a variety of developmental processes in *Arabidopsis*. For example, the *AtCHR2* (*BRM*) was involved in morphological traits of leaves and roots as well as reproduction [27, 28, 31, 32, 60]. The stem cell pool maintenance of the apical meristem was controlled by *AtCHR3* (SYD) [23]. The brassinosteroid and gibberellin signaling pathways were regulated by *AtCHR6* (*PICKLE*) during skotomorphogenic growth [42]. Furthermore, AtCHR2 (AtBRM) also acts as a positive regulator in GA biosynthesis, which regulates GA-responsive genes in a DELLA-independent manner [61]. *AtCHR13* (*PIE*) and *AtCHR1* (*DDM1*) are involved in DNA repair and DNA methylation [62, 63]. A recent study in tomato showed that constitutively overexpressed *SlCHR8* caused significantly shorter roots and hypocotyls with reduced cotyledon size in transgenic tomato plants (cv. Micro-Tom) [47]. In this study, we found that many protein motifs such as motifs 13, 16 20, 7, 8, and 18 in the DRD1 subfamily and motifs 17, 14, 15, and 19 in the Rad5/16-like family are unique to or mainly exist in one group of *SlCHRs* (Table 3), indicating that the same group *SlCHRs* may play similar roles as their *Arabidopsis* counterparts. The expression profiles of *SlCHRs* indicated that some *SlCHRs* may play different roles compared with their homologs in *Arabidopsis*. For instance, *SlCHR8* was mainly expressed in roots and fruits (Figure 6(a)), indicating that it may function in root and fruit development, which is consistent with the report that overexpression of *SlCHR8* in tomato resulted in considerably compacter growth including significantly shorter roots and hypocotyls as well as reduced cotyledon and fruit size [47]. In contrast, its *Arabidopsis* homolog *BRM* (*AtCHR2*) functions in leaf and flower development [27, 31, 60]. Furthermore, functional divergence was observed between *SlCHR41* and its homolog *AtCHR3* (*SYD*), since *SlCHR41* is poorly expressed in flowers while *AtCHR3* is highly expressed in this organ (Figure 6(a)). Both *SlCHR4* and *SlCHR5* show a peak expression in buds (Figure 6(c)), indicating a role in gamete and/or flower development. In addition, we found that some *SlCHRs* respond to environmental stimuli. For instance, the expression of most *SlCHRs* is repressed by SA but enhanced by ABA (Figure 7). In *Arabidopsis*, CHR2 (BRM) is involved in the ABA signaling pathway via binding the regulatory regions of *ABI3* and *ABI5* genes [26]. Further Chip-seq analyses show that BRM-activated genes were primarily enriched in the categories of jasmonic acid and gibberellic acid responses, while BRM-repressed genes were primarily enriched in the categories of salicylic acid and light responses [64]. Collectively, these data indicated the importance of CHRs in plants. Further research is required to investigate the molecular mechanism on how SICHRs are involved in tomato development and hormone signaling pathways.

## 5. Conclusions

In this study, a total of 45 full-length SlCHRs were identified in tomato, which are clustered into 6 groups. Most SlCHRs within a group are highly conserved in sequence features, gene structures, and motifs, suggesting the functional conservation of SlCHRs within a group. Furthermore, diversities in the specific domains identified in different groups indicate that some SlCHRs may have undergone functional diversification. The expression profiles suggest that most *SlCHRs* are expressed constitutively in tomato organs, and RT-qPCR analyses show that the expression of some *SlCHRs* is modulated by the exogenous stimuli, suggesting that *SlCHRs* may play important roles in plant development and stress responses.

## Figures and Tables

**Table 1:** Snf2 family genes in tomato and *Arabidopsis*.

Group	Subfamily	*Arabidopsis thaliana*	*Solanum lycopersicum*	Loc. symbol
Snf2-like	Chd1	CHR5	SlCHR45	Solyc12g099910
	Mi-2	CHR6 (PICKLE)	SlCHR27	Solyc06g054560
		CHR4	SlCHR33	Solyc08g029120
		CHR7		
	CHD7			
	Iswi	CHR11	SlCHR2	Solyc01g067390
		CHR17	SlCHR26	Solyc06g050510
	Lsh	CHR1 (DDM1)	SlCHR14	Solyc02g062780
			SlCHR13	Solyc02g085390
	Snf2	CHR2 (BRM)	SlCHR8	Solyc01g079690
		CHR3 (SYD)	SlCHR41	Solyc11g062010
		CHR12	SlCHR6	Solyc01g094800
		CHR23		
	ALC1			
Swr1-like	Swr1	CHR13 (PIE)	SlCHR17	Solyc03g063220
	Ino80	CHR21 (Ino80)	SlCHR19	Solyc04g016370
	Etl1	CHR19	SlCHR10	Solyc02g014770
	EP400	CHR10	SlCHR7	Solyc01g090650
SSO1653-like	Mot1	CHR16	SlCHR36	Solyc08g074500
	ERCC6	CHR8	SlCHR39	Solyc09g066480
		CHR24	SlCHR3	Solyc01g068280
	SSO1653			
Rad54-like	Rad54	CHR25 (RAD54)	SlCHR22	Solyc04g056400
		CHR9	SlCHR32	Solyc07g053870
	Arip4			
	ATRX	CHR20	SlCHR20	Solyc04g050150
	JBP2	CHR38	SlCHR25	Solyc05g044510
		CHR42		
	DRD1	CHR35 (DRD1)	SlCHR9	Solyc01g109970
		CHR34	SlCHR34	Solyc08g061410
		CHR31	SlCHR35	Solyc08g062000
		CHR40	SlCHR11	Solyc02g033050
			SlCHR37	Solyc08g077610
			SlCHR21	Solyc04g054440
			SlCHR1	Solyc01g060460
			SlCHR38	Solyc08g077690
			SlCHR4	Solyc01g068300
			SlCHR5	Solyc01g068320
Distant	SMARCAL1	CHR14	SlCHR18	Solyc03g115520
		CHR18	SlCHR44	Solyc12g098860
Rad5/16-like	SHPRH	CHR39	SlCHR40	Solyc11g005250
		CHR36		
	Lodestar			
	Ris1	CHR30	SlCHR12	Solyc02g050280
		CHR33	SlCHR16	Solyc03g006570
		CHR27	SlCHR24	Solyc05g044480
		CHR28	SlCHR23	Solyc04g056410
		CHR26	SlCHR31	Solyc07g052100
		CHR37	SlCHR30	Solyc07g051970
		CHR41	SlCHR28	Solyc06g065730
			SlCHR29	Solyc07g051960
	Rad5/16	CHR32	SlCHR15	Solyc03g005460
		CHR29	SlCHR42	Solyc11g066790
		CHR22	SlCHR43	Solyc12g020110

**Table 2:** The nonsynonymous substitution (Ks) of SlCHR paralogous genes.

Paralogous genes	Ks
*SlCHR27* (Chr6)/*SlCHR33* (Chr8)	1.030
*SlCHR2* (Chr1)/*SlCHR26* (Chr6)	1.360
*SlCHR6* (Chr1)/*SlCHR8* (Chr1)	0.860
*SlCHR13* (Chr2)/*SlCHR14* (Chr2)	0.172
*SlCHR7* (Chr1)/*SlCHR19* (Chr4)	0.840
*SlCHR10* (Chr2)/*SlCHR17* (Chr3)	1.500
*SlCHR34* (Chr8)/*SlCHR35* (Chr8)	0.285
*SlCHR11* (Chr2)/*SlCHR37* (Chr8)	1.085
*SlCHR1* (Chr1)/*SlCHR38* (Chr8)	0.177
*SlCHR4* (Chr1)/*SlCHR5* (Chr1)	0.107
*SlCHR3* (Chr1)/*SlCHR39* (Chr9)	1.290
*SlCHR22* (Chr4)/*SlCHR32* (Chr7)	0.729
*SlCHR15* (Chr3)/*SlCHR42* (Chr11)	0.550
*SlCHR12* (Chr2)/*SlCHR16* (Chr3)	1.465
*SlCHR23* (Chr4)/*SlCHR31* (Chr7)	1.238
*SlCHR28* (Chr6)/*SlCHR29* (Chr7)	1.346

**Table 3:** Schematic distribution of conserved motifs of SlCHRs.

													Helicase_C domain				
SlCHR45		**12**	**3**	**5**			**9**	2	14			**6**	10	4	1	**11**	
SlCHR27		**12**	**3**	**5**			**9**	2	14			**6**	10	4	1	**11**	
SlCHR33		**12**	**3**	**5**			**9**	2	14			**6**	10	4	1		
SlCHR2		**12**	**3**	**5**			**9**	2	14			**6**	10	4	1	**11**	
SlCHR26		**12**	**3**	**5**			**9**	2	14			**6**	10	4	1	**11**	
SlCHR14		**12**	**3**	**5**			**9**	2	14			**6**	10	4	1	**11**	
SlCHR13		**12**	**3**	**5**			**9**	2	14			**6**	10	4	1	**11**	
SlCHR8		**12**	**3**	**5**			**9**	2	14			**6**	10	4	1		
SlCHR41		**12**	**3**	**5**			**9**	2	14			**6**	10	4	1	**11**	
SlCHR6		**12**	**3**	**5**			**9**	2	14			**6**	10	4	1	**11**	
SlCHR17		**12**	**3**	**5**			**9**	2	14			**6**	10	4	1	**11**	
SlCHR19		**12**	**3**	**5**			**9**	2	14			**6**	10	4	1	**11**	
SlCHR10		**12**	**3**	**5**			**9**	2	14			**6**	10	4	1	**11**	
SlCHR7		**12**	**3**	**5**			**9**	2	14			**6**	10	4	1	**11**	
SlCHR36		**12**	**3**	**5**			**9**	2	14			**6**	10	4	1	**11**	
SlCHR39		**12**	**3**	**5**			**9**	2	14			**6**	10	4	1	**11**	
SlCHR3		**12**	**3**	**5**			**9**	2	14			**6**	10	4	1	**11**	
SlCHR22												**6**	10	4	1	**11**	
SlCHR32		**12**	**3**	**5**			**9**	2	14			**6**	10	4	1	**11**	
SlCHR20		**12**	**3**	**5**			**9**	2	14			**6**	10	4	1	**11**	
SlCHR25			**3**	**5**			**9**	2	14			**6**	10	4	1	**11**	
SlCHR9		**12**	**3**	**5**			**9**	2	14			**6**	10	4	1	**11**	
SlCHR34			**3**	**5**	**16**			2	7	8		**6**	10	4	1	**11**	18
SlCHR35		**12**			**16**	**20**		2		8		**6**		4	1		
SlCHR11	**13**	**12**	**3**	**5**	**16**	**20**	**9**	2	7	8		**6**	10	4	1	**11**	18
SlCHR37	**13**	**12**	**3**	**5**	**16**	**20**	**9**	2	7	8		**6**	10	4	1	**11**	18
SlCHR21	**13**	**12**	**3**	**5**	**16**	**20**	**9**	2	7	8		**6**	10	4	1	**11**	18
SlCHR1	**13**	**12**	**3**	**5**	**16**	**20**	**9**	2	7	8		**6**	10	4	1	**11**	18
SlCHR38	**13**	**12**	**3**	**5**	**16**	**20**	**9**	2	7	8		**6**	10	4	1	**11**	18
SlCHR4								2	7	8		**6**	10	4	1	**11**	18
SlCHR5	**13**	**12**	**3**	**5**	**16**	**20**	**9**	2	7	8		**6**	10	4	1	**11**	18
SlCHR44			**3**	**5**			**9**		14			**6**	10	4	1	**11**	
SlCHR18		**12**	**3**	**5**			**9**		14			**6**	10	4	1		
SlCHR40		**12**	**3**	**5**			**9**				19	**6**			1		
SlCHR12		**12**	**3**	**5**	17		**9**	2	14	15	19	**6**	10	4	1	**11**	
SlCHR16		**12**	**3**	**5**	17		**9**	2	14	15	19	**6**	10	4	1	**11**	
SlCHR24		**12**	**3**	**5**	17		**9**	2	14	15	19	**6**	10	4	1	**11**	
SlCHR23		**12**	**3**	**5**	17		**9**	2	14	15	19	**6**	10	4	1	**11**	
SlCHR31		**12**	**3**	**5**	17		**9**	2	14	15	19	**6**	10	4	1	**11**	
SlCHR30		**12**	**3**	**5**	17		**9**	2	14	15	19	**6**	10	4	1		
SlCHR28		**12**	**3**	**5**	17		**9**	2	14	15	19	**6**	10	4	1		
SlCHR29		**12**	**3**	**5**	17		**9**	2	14				10	4	1		
SlCHR15		**12**	**3**	**5**	17		**9**	2	14	15	19		10	4	1	**11**	
SlCHR42		**12**	**3**	**5**	17		**9**	2	14	15	19		10	4	1	**11**	
SlCHR43		**12**	**3**	**5**	17		**9**	2	14	15	19	**6**	10	4	1	**11**	

## Data Availability

The original data of Snf2-like family proteins are available from ChromDB (http://www.chromdb.org). The sequences of tomato CHR proteins are available from the International Tomato Genome Sequencing Project (https://solgenomics.net/organism/Solanum_lycopersicum/genome).

## Supplementary Materials

Supplementary Materials Supplemental Table 1: sequence features of SlSnf2s in tomato and Arabidopsis. Gene IDs, protein length, intron number, pI, and molecular weight of Snf2s in *A. thaliana* and tomato are shown. Supplemental Table 2: primers used in this study. Supplemental Figure 1: phylogenetic tree of SlCHR proteins. Maximum likelihood phylogenetic tree of predicted proteins from tomato. Bootstrap values higher than 50% are shown. Supplemental Figure 2: neighbor-joining (NJ) phylogenetic tree for Snf2s in thaliana (At), rice (Os), and tomato (Sl). The groups of homologous genes identified and bootstrap values are shown. The reliability of branching was assessed by the bootstrap resampling method using 1,000 bootstrap replicates. Supplemental Figure 3: exon-intron structures of SlCHRs. Introns are represented by lines. Exons are indicated by green boxes. Intron phases are shown by 0, 1, and 2. Supplemental Figure 4: the distribution of intron phases in Snf2s in *A. thaliana* and tomato. Supplemental Figure 5: sequence comparison of HSA domain of Snf2 subfamily in plant, yeast, and human. The conserved amino acid residues are marked in red. Supplemental Figure 6: sequence comparison of helicase_C domain of SlCHRs. The motifs of 10, 4, and 1 are shown. Supplemental Figure 7: sequence logo of the helicase_C domain of SlCHRs.
